# Vesicovaginal Fistula Robotics-Assisted Repair: A Systematic Review and Quantitative Synthesis

**DOI:** 10.1007/s00192-026-06578-8

**Published:** 2026-04-02

**Authors:** Miguel Tavares, Maria do Carmo Pinto, Gonçalo Conde Carvalho, Miguel Silva-Ramos

**Affiliations:** 1 Faculty of Medicine, Institute for the Biomedical Sciences Abel Salazar, Porto, Portugal; 2 Serviço de Urologia, Hospital Dr. Nélio Mendonça, SESARAM, EPERAM—Serviço de Saúde da Região Autónoma da Madeira, Avenida Luís de Camões, n°57, 9004-514 Funchal, Portugal; 3 Unidade Local de Saúde de Santo António, Porto, Portugal; 4 Instituto de Ciências Biomédicas Abel Salazar (ICBAS), Universidade do Porto, Porto, Portugal

**Keywords:** Extravesical, Robotics-assisted surgery, Transabdominal repair, Transvaginal repair, Transvesical, Vesicovaginal fistula

## Abstract

**Introduction and Hypothesis:**

The aim of this review is to evaluate the current literature on robotics-assisted vesicovaginal fistula (VVF) repair, with a focus on comparing outcomes of two approaches: transvesical and extravesical.

**Methods:**

Following Preferred Reporting Items for Systematic Reviews and Meta-Analyses guidelines, PubMed, Scopus, and Web of Science were screened from inception to 13 April 2025, using variations of the search items: “vesicovaginal fistula” and “robotic surgery.” Comparative studies and case series with *n* ≥ 5 were eligible. Outcomes were operative time (OT), estimated blood loss (EBL), postoperative complication (POC), recurrence rate, length of hospitalization (LOS), and urethral catheterization time (CT). Statistical analysis was conducted to determine overall and subgroup-specific outcomes and compare techniques for statistically significant differences.

**Results:**

Fourteen studies (206 patients) met the inclusion criteria. Combined mean OT was 169.0 ± 69.5 min, with minimal blood loss across all studies (14–90 ml). Overall POC and recurrence rates were low (4.37% and 2.91% respectively), with major complications at 0.97%. No significant differences were found between surgical approaches. Most studies reported hospital stays under 5 days, although CTs varied. Two case series on recurrent VVF reported 100% success, no complications or recurrences, low blood loss, and shorter OTs, supporting the safety and efficacy of robotics-assisted repair in complex cases.

**Conclusions:**

Robotics-assisted VVF repair was found to be a safe, effective, reproducible technique, with low complication and recurrence rates, minimal blood loss, and short hospital stays. No statistically significant difference was found between techniques. The findings also support robotics-assisted surgery as an especially valuable option in recurrent cases.

## Introduction

A vesicovaginal fistula (VVF) is an abnormal communication between the bladder and the vagina and it is the most common type of urinary fistula. It results in continuous involuntary urinary leakage, severely impacting patients’ physical, emotional, and social well-being [1]. Although prolonged labor and cesarean section remain the leading causes of VVF in low-resource settings, iatrogenic injury during gynecological surgeries, particularly hysterectomy, is the primary etiology in developed countries [1].

Conservative management strategies, such as prolonged urinary diversion (through a Foley catheter) and fulguration, have been described for small, early-presenting fistulas [2]. However, spontaneous closure is rare, and most patients require surgical repair to achieve definitive resolution [2, 3]. There are two main surgical approaches for VVF: transvaginal (TV) and transabdominal (TA). Although the former is effective in most patients with VVF requiring surgery, it is typically favored in small, low-lying fistulas [3]. Even though TV repair is the less invasive approach, the TA route has its own advantages, such as the ability to place interposition tissue between the suture lines or to perform concomitant abdominal procedures (such as ureteral reimplantation and augmentation cystoplasty), and decreases the risk of vaginal shortening, which is sometimes associated with the vaginal approach [4]. As such, some common indications for the TA approach are a narrow vagina, supratrigonal fistulas, recurrent fistulas after failed TV repairs, or when a concomitant abdominal procedure is needed. It is also especially well-suited to large (> 3-cm), recurrent, and multiple fistulas in difficult locations (e.g., in close proximity to the ureters) [1, 4].

Open surgery has historically been the gold standard for TA repairs, but in recent decades, minimally invasive techniques, particularly laparoscopy, have gained popularity by offering reduced perioperative morbidity and faster recovery. Nevertheless, laparoscopy poses technical challenges, especially in the deep pelvis [5, 6]. Limitations of the laparoscopic approach described by many authors are its steep learning curve, complex intracorporeal suturing skill requirements, and inherent difficulty in dissecting between the bladder and the vagina [7, 8]. The advantages associated with robotics-assisted surgery (RAS) allow for more precise dissection and suturing, while being associated with a more favorable learning curve, mitigating the limitations of the open and laparoscopic TA approaches [4, 8, 9].

Two main approaches to accessing the fistula can be utilized, the TV and the extravesical (EV). The TV approach involves a cystotomy for easy access to the fistula through the direct intravesical visualization of the fistulous orifice. In contrast, EV repair avoids opening the bladder by accessing the fistula through dissection of the vesicovaginal space [7, 10], causing less bladder trauma and potentially reducing postoperative bladder irritability and risk of bladder-related complications [5, 11]. However, it provides limited exposure in cases of complex, large, or trigonal fistulas, potentially making dissection and closure more difficult than the TV approach. The classic TV approach, originally described by O’Conor and Sokol, involves bivalving the bladder [12]. More recently, the preferred technique has shifted toward the Mini O’Conor approach, which utilizes a limited posterior cystotomy near the fistula site. This modification is associated with reduced operative time (OT), lower blood loss, and decreased postoperative bladder spasms [8, 13]. In this review, studies employing either the classic or the Mini O’Conor technique were grouped under the TV approach.

This systematic review and quantitative synthesis are aimed at synthesizing the current evidence on robotics-assisted VVF repair, with a specific focus on comparing perioperative and postoperative outcomes of the TV and EV techniques.

## Materials and Methods

### Search Strategy

In accordance with the Preferred Reporting Items for Systematic Reviews and Meta-Analyses (PRISMA) Guidelines [14], a search of the literature was conducted in three databases (PubMed, Scopus, and Web of Science), from inception to 13 April 2025, using combinations of the following keywords and their respective MeSH/Emtree terms: “vesicovaginal fistula”; “VVF”; “gynecological fistula”; “robotic surgical procedures”; “robot-assisted”; “robotic”; “robot”. The reference lists of the studies included were also considered in order to identify other potentially eligible studies.

### Article Selection

Comparative and noncomparative prospective studies, retrospective studies, and case series with a population size ≥ 5 were included. Reviews, smaller case series and case reports were excluded. The population included consisted of adult patients (≥ 18 years) who underwent repair of non-oncological VVFs using a robotics-assisted approach. The intervention was repair of VVFs through TA RAS and all repair approaches (TV and EV) were included. Studies in which the surgical approach was not clearly described were excluded. Studies in which other intraoperative procedures (in addition to fistula repair) were performed were included. Comparison of outcomes was made between the TV and EV surgical approaches. The evaluated outcomes were OT, estimated blood loss (EBL), postoperative complication (POC) rate (according to the Clavien–Dindo Classification System) [15], recurrence rate, hospital length of stay (LOS), and urethral catheterization time (CT). A risk of bias assessment was conducted for each selected article, using the Joanna Briggs Institute (JBI) Checklist for Case Series and the ROBINS-I V2 tool for comparative studies [16, 17]. Screening, selection of studies, and risk-of-bias assessment were conducted by two reviewers independently, with disagreements resolved by a third.

**Fig. 1:**
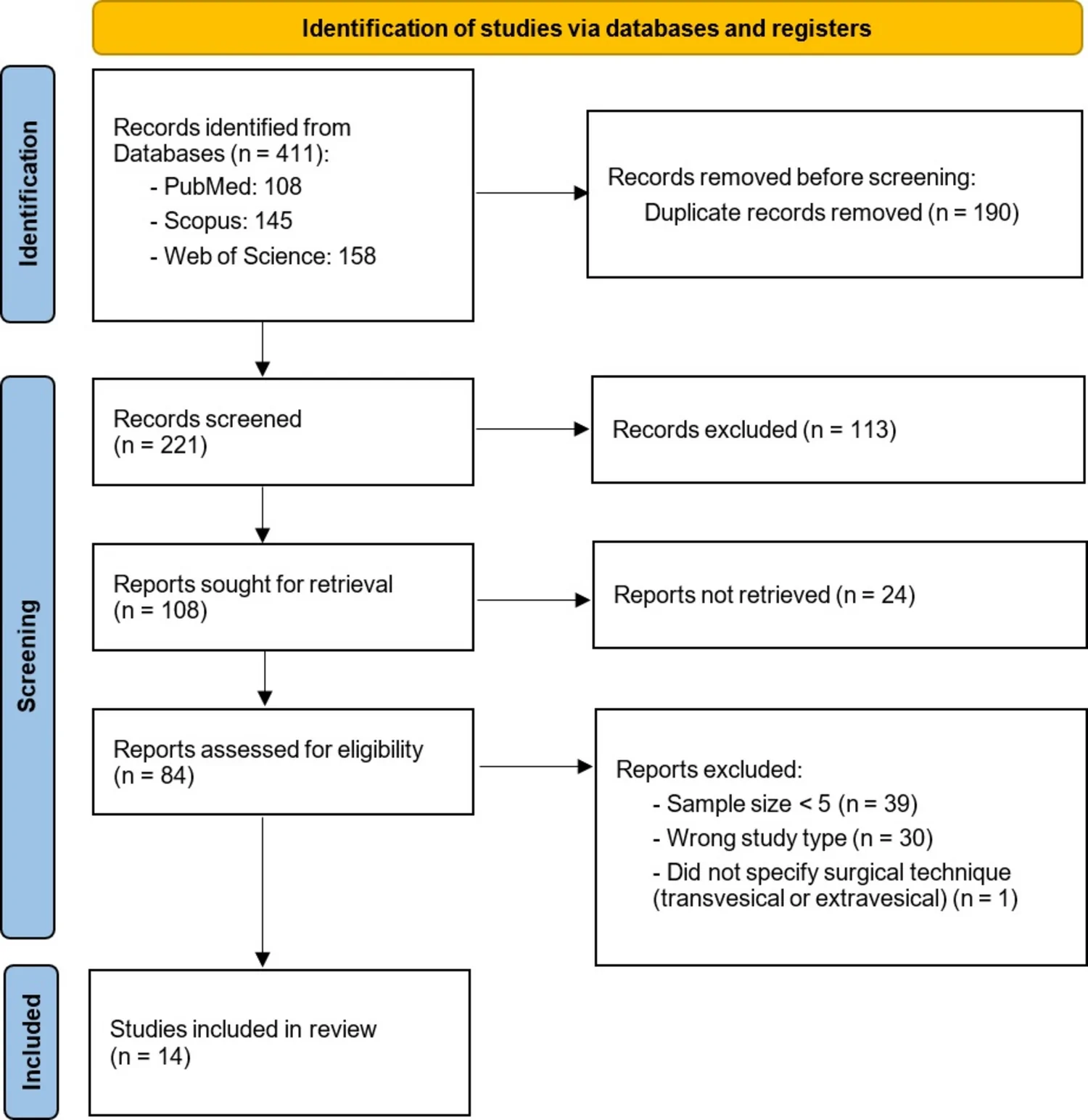
Preferred Reporting Items for Systematic Reviews and Meta-Analyses (PRISMA) flow diagram for study selection

The initial search resulted in 411 records (PubMed: 108, Scopus: 145, Web of Science: 158), which amounted to 221 after duplicate removal. A screening process based on title and abstract resulted in 108 full texts being sought for retrieval (*n* = 113 excluded). Of these, a total of 84 full-text articles were successfully retrieved for eligibility assessment. In total, 14 were included in the review, based on the inclusion/exclusion criteria described. Thirty-nine studies were excluded for having sample sizes of less than 5 patients, 30 studies were excluded for being a study type other than those accepted by the criteria and because it did not report on the surgical approach to VVF repair (TV or EV). A PRISMA flow diagram representing the study selection process is presented in Fig. 1.

### Data Synthesis

Given the heterogeneity of study designs, patient populations, surgical techniques, and outcome reporting, as well as the small sample sizes of the studies included, a formal meta-analysis was not performed. Instead, a descriptive synthesis of the available data was undertaken, with exploratory comparisons performed where sufficient data were available.

### Statistical Analysis

A combined mean and standard deviation (SD) were calculated for each technical group (TV and EV) for the quantitative variable, the OT. The combined mean was calculated by multiplying the mean of each study with its respective sample size and then dividing the sum of the result by the total number of patients in the studies included, per technique subgroup. The combined SD per group was achieved using Cochrane's formula for combining groups [18]. Thereafter, the combined means and SDs of both groups were compared regarding OT using Student’s *t* test for unpaired samples. Regarding the qualitative variables—POC and recurrence rates—Fisher’s exact test was used to calculate statistical significance between the two techniques, as more than 20% of the expected frequencies of this data set were less than 5. A *p* value < 0.05 was considered statistically significant. Because individual patient-level data were unavailable for the majority of the studies included and were often incomplete across reported variables, analyses were necessarily based on aggregated study-level summaries from heterogeneous retrospective series. As a result, formal assumptions of normality could not be verified, and intergroup comparisons using Student’s *t* test should be considered exploratory and interpreted with caution. Although several studies reported medians with associated measures of dispersion, no transformation of medians to means or estimation of SDs was performed. Such conversions would have required subsequent sensitivity analyses to assess the robustness of the results; however, given the small sample sizes and substantial clinical heterogeneity across studies, performing these transformations was considered methodologically inappropriate and was therefore avoided. Consequently, no statistical analysis was carried out to compare EBL between groups as it was minimal in all studies and reported heterogeneously among them (11 studies reported the mean, but only 6 [4, 19–23] provided the SD or enough data to calculate it, with only one study [23] in the EV group with a *n* = 5). Likewise, no statistical analysis was performed regarding LOS and CT, as these were mostly reported without their mean and SD values, which hinders technique subgroup comparison.

### Ethical Considerations

Ethical approval was not required for this study, as it is a systematic review of previously published literature and does not involve human participants, new patient data, or identifiable personal information.

## Results

### Characteristics of the Studies Included

Fourteen studies were included in the review, totaling 206 patients. The oldest study was published in 2006 [24], whereas the most recent one was published in 2025 [20]. Population size varied from 5 [19, 23, 24] to 33 [22] patients. Twelve studies were retrospective case series and 2 were nonrandomized retrospective comparative studies [9, 25]. The TV approach to VVF surgical repair was used in 10 studies [4, 8, 9, 13, 19–22, 24, 26], and the EV approach in another 3 [23, 27, 28]; 1 comparative study compared the two approaches [25]. In the overall review, 169 patients underwent a TV approach and 37 an EV approach. According to the Oxford Centre for Evidence-Based Medicine 2011 [29], the 12 case series included have a level of evidence of 4 and the 2 comparative studies a level of evidence of 3b. The application of the JBI Critical Appraisal Checklist for Case-Series revealed some inconsistencies in the reporting of fistula characteristics, namely fistula size [4, 23, 26, 27], as well as in patient characteristics [21, 27] and outcome measures [28]. One comparative study [9] had a “moderate” risk of bias, and the other had a “serious” risk of bias, on account of not having controlled for any confounding factors [25]. The 14 studies included and their characteristics are represented in Table 1; risk-of-bias assessment is represented in Tables 2 and 3.

**Table 1:** Characteristics of the studies included

Reference	Journal	*n*	Surgical technique	Type of study	Level of evidence
Sundaram et al. [24]	*Urology*	5	Transvesical	Retrospective case series	Level 4
Hemal et al. [21]	*Journal of Urology*	7	Transvesical	Retrospective case series	Level 4
Gupta et al. [9]	*Journal of Endourology*	12	Transvesical	Retrospective nonrandomized comparative study	Level 3b
Gellhaus et al. [27]	*BJU International*	10	Extravesical	Retrospective case series	Level 4
Agrawal et al. [4]	*Urology*	10	Transvesical	Retrospective case series	Level 4
Matei et al. [19]	*Urologia Internationalis*	5	Transvesical	Retrospective case series	Level 4
Bora et al. [13]	*International Urogynecology Journal*	30	Transvesical	Retrospective case series	Level 4
Sanderson et al. [23]	*JSLS*	5	Extravesical	Retrospective case series	Level 4
Körner et al. [28]	*Scandinavian Journal of Urology*	13	Extravesical	Retrospective case series	Level 4
Chandna et al. [22]	*Urology*	33	Transvesical	Retrospective case series	Level 4
Kumar et al. [8]	*Urology Annals*	15	Transvesical	Retrospective case series	Level 4
Quadri et al. [26]	*Journal of Clinical and Diagnostic Research*	24	Transvesical	Retrospective case series	Level 4
Lecoanet et al. [25]	*International Urogynecology Journal*	22	Transvesical (13)	Retrospective nonrandomized comparative study	Level 3b
			Extravesical (9)		
Yang et al. [20]	*Archives of Gynecology and Obstetrics*	15	Transvesical	Retrospective case series	Level 4

**Table 2:**
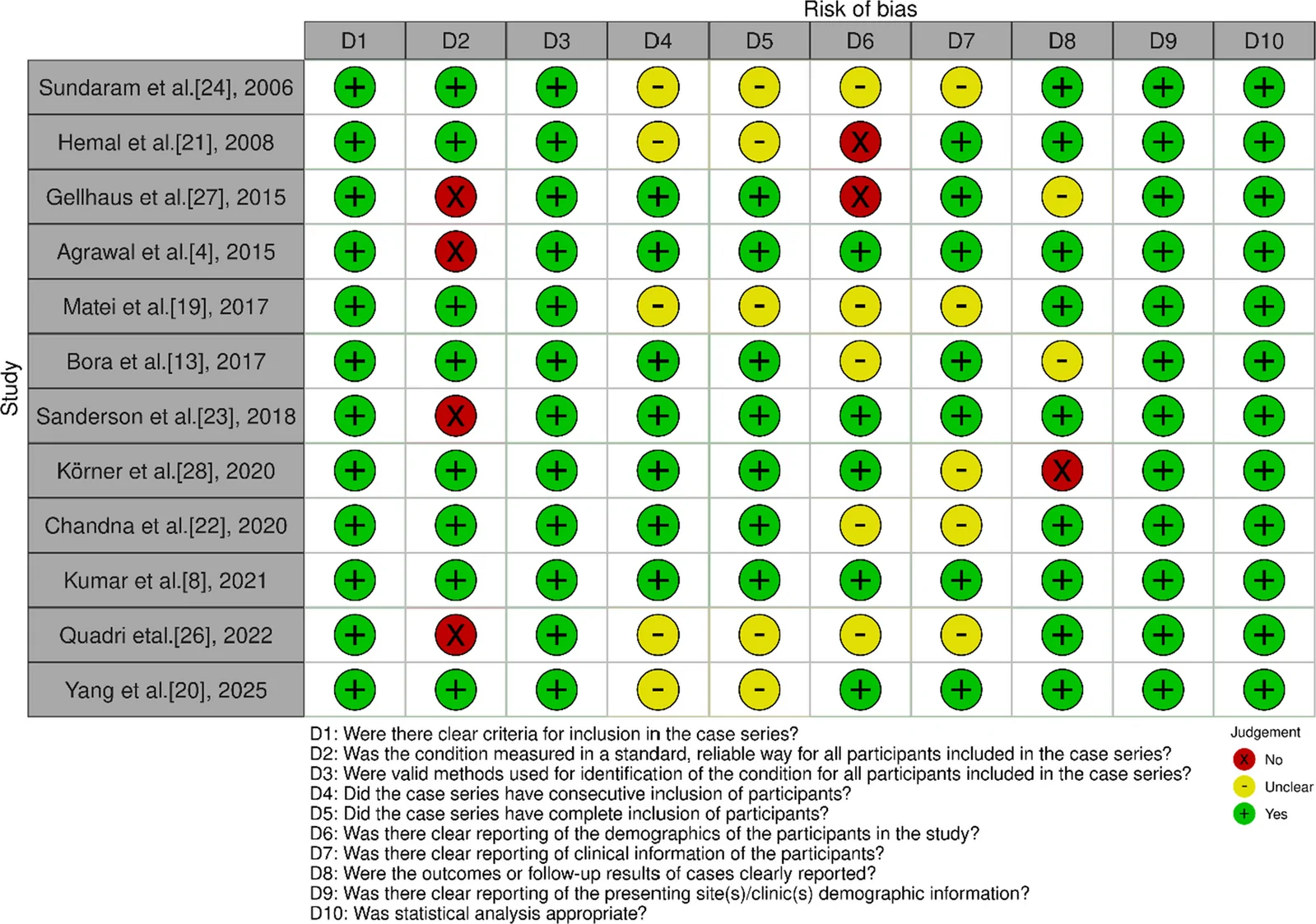
Application of JBI critical appraisal checklist for case-series

**Table 3:**
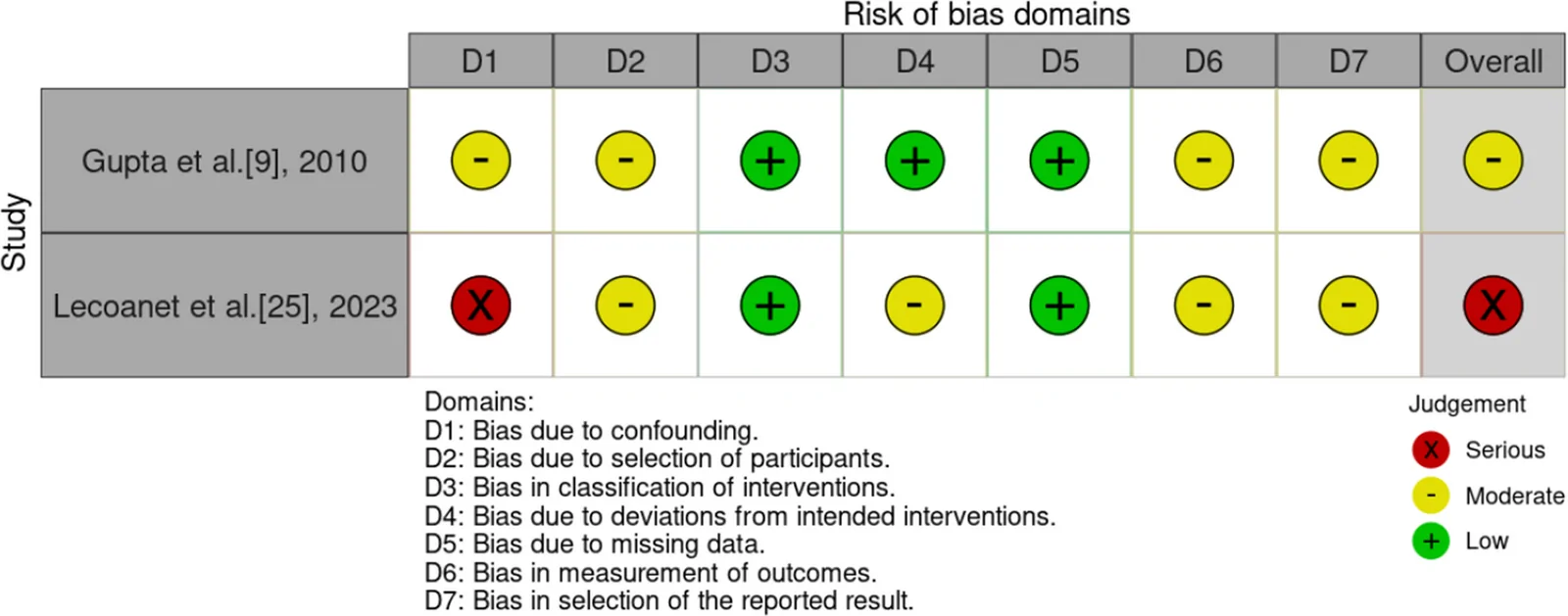
Application of ROBINS-I Tool for non-randomized studies

### Patient, Fistula, and Procedural Characteristics

The average age of the patient population varied from 27.1 to 62 years old [9, 19]. Their body mass index varied from 24.3 to 32.4 kg/m^2^ [8, 23]. The most common etiology of the fistula was post-hysterectomy (*n* = 138), followed by obstetrical causes (*n* = 36), such as obstructed labor or cesarean section. In one case series the etiology was post-radical oncological surgery for uterine or ovarian carcinoma; however, fistula repair surgery was conducted “(...) after an adequate follow-up period confirming no cancer relapse (...)” [19]. Regarding fistula characteristics, mean fistula size varied between 5 and 31 mm [19, 24], whereas location was mostly supratrigonal, with only one case series [19] exclusively presenting trigonal fistulas, and in total only 17 out of 196 fistulas were trigonal. Two studies reported specifically on recurrent fistula repair [9, 21], whereas four others reported on primary fistula repair only [19, 23, 24, 28]; the rest were mixed. Time from presentation/index surgery to repair varied from 2.6 to 10.2 months [23, 27]. Six case series described additional procedures performed intraoperatively at the same time as VVF repair [4, 8, 20, 22, 26, 27]. Most studies used some type of tissue interposition (omentum, peritoneum, colonic epiploicae, fat pads, and bladder adventitia), whereas only one case series [19] did not use interposition tissue in any patient and two other studies did not interpose any tissue in a significant proportion of their patients (12 out of 30) [13] and (7 out of 33) [22]. The described patient, fistula, and procedural characteristics are represented in Tables 4 and 5.

**Table 4:** Patient and fistula characteristics per study and in the whole review

Reference	*n*	Mean age (years)	Mean BMI (kg/m^2^)	Etiology	Mean fistula size (mm)	Fistula location	Recurrent fistulas	Mean time from index surgery/symptoms to repair (months)
Sundaram et al. [24]	5	26–58	—	Hysterectomy (*n* = 4)	31 ± 8.9	Supratrigonal (*n* = 5)	Primary (*n* = 5)	**–**
				Other surgeries (*n* = 1)				
Hemal et al. [21]	7	—	—	Hysterectomy (*n* = 3)	30 ± 6.6	Supratrigonal (*n* = 7)	Recurrent (*n* = 7)	7.1 ± 2.7
				Obstetric (*n* = 4)				
Gupta et al. [9]	12	27.1 (16–46)	—	Obstetric (*n* = 8)	28 (15–60)	Supratrigonal (*n* = 12)	Recurrent (*n* = 12)	6 (3–14)
				Hysterectomy (*n* = 4)				
Gellhaus et al. [27]	10	—	—	Obstetric (*n* = 10)	—	—	Primary (*n* = 8)	10.2 (1–68)
							Recurrent (*n* = 2)	
Agrawal et al. [4]	10	45.2 ± 8.8	30.2 ± 6.6	Hysterectomy (*n* = 10)	—	Supratrigonal (*n* = 10)	Primary (*n* = 7)	8.7 ± 12.2
							Recurrent (*n* = 3)	
Matei et al. [19]	5	62.2 ± 6.2	—	Other surgeries (*n* = 5)	5.2 ± 2.3	Trigonal (*n* = 5)	Primary (*n* = 5)	—
Bora et al. [13]	30	43.5 ± 8.6	—	Hysterectomy (*n* = 27)	10.4 ± 7	Supratrigonal (*n* = 28)	Primary (*n* = 21)	—
				Obstetric (*n* = 2)				
				Unknown (*n* = 1)		Trigonal (*n* = 2)	Recurrent (*n* = 9)	
Sanderson et al. [23]	5	51.8 ± 9.3	32.4 ± 1.1	Hysterectomy (*n* = 4)	—	Supratrigonal (*n* = 5)	Primary (*n* = 5)	2.6 ± 0.98
				Other surgeries (*n* = 1)				
Körner et al. [28]	13	45 ± 14.5	28 ± 5.2	Hysterectomy (*n* = 10)	13.2 ± 10.3	Supratrigonal (*n* = 11)	Primary (*n* = 13)	—
				Obstetric (*n* = 1)				
				Foreign body (*n* = 1)				
				Other surgeries (*n* = 1)		Trigonal (*n* = 2)		
Chandna et al. [22]	33	42.7 ± 7.2	—	Hysterectomy (*n* = 27)	12.8 ± 9.2	Supratrigonal (*n* = 30) Trigonal(*n* = 3)	Recurrent (*n* = 21) Primary(*n* = 12)	—
				Obstetric (*n* = 5)				
				Radiation (*n* = 1)				
Kumar et al. [8]	15	37.27 (26–45)	(16.7–30.1)	Hysterectomy (*n* = 13)	12.2 (5–35)	Supratrigonal (*n* = 15)	Primary (*n* = 12)	5.4 (2–36)
				Obstetric (*n* = 2)			Recurrent (*n* = 3)	
Quadri et al. [26]	24	40.33 ± 5.73	—	Hysterectomy (*n* = 24)	—	Supratrigonal (*n* = 24)	Primary (*n* = 19)	—
							Recurrent (*n* = 5)	
Lecoanet et al. [25]								
Transvesical	13	—	26.3 ± 7.5	Other surgeries (*n* = 11)	—	Supratrigonal (*n* = 9)	Primary (*n* = 9)	—
				Obstetric (*n* = 1)				
				Trauma (*n* = 1)		Trigonal (*n* = 4)	Recurrent (*n* = 4)	
Extravesical	9	—	27.3 ± 9.7	Other surgeries (*n* = 6)	—	Supratrigonal (*n* = 9)	Primary (*n* = 8)	
				Obstetric (*n* = 3)			Recurrent (*n* = 1)	
Yang et al. [20]	15	45.2 ± 12.7	24.7 ± 4.1	Hysterectomy (*n* = 12)	17.0 ± 5.0	Supratrigonal (*n* = 14)	Primary (*n* = 8)	6.9 ± 3.23
				Foreign body (*n* = 2)				
				Other Surgeries (*n* = 1)		Trigonal (*n* = 1)	Recurrent (*n* = 7)	
Whole review	206	42.29 (*n* = 162)	26.88 (*n* = 80)	Hysterectomy (*n* = 138)	15.3 (*n* = 135)	Supratrigonal (*n* = 179)	Primary (*n* = 132)	6.89 (*n* = 74)
				Obstetric (*n* = 36)				
				Other surgeries (*n* = 26)				
				Foreign body (*n* = 3)				
				Trauma (*n* = 1)				
				Radiation (*n* = 1)		Trigonal (*n* = 17)		
				Unknown (*n* = 1)		Unknown (*n* = 10)	Recurrent (*n* = 74)	

*BMI* body mass index,

**Table 5:** Procedural characteristics per study and in the whole review

		*n*	Surgical Technique	Concomitant Procedures	Tissue Interposition
Sundaram et al. 2006 [24]		5	Transvesical (5)	—	Omentum (5)
Hemal et al. 2008 [21]		7	Transvesical (7)	—	Other Tissues (7)
Gupta et al. 2010 [9]		12	Transvesical (12)	—	Omentum (7) Peritoneum (3) Sigmoid Epiploicae (2)
Gellhaus et al. 2015 [27]		10	Extravesical (10)	Ureteric reimplantation (1)	Unknown (5) Omentum (4) Other Tissues (1)
Agrawal et al. 2015 [4]		10	Transvesical (10)	Ureteric reimplantation (3) Sigmoid colectomy (1)	Sigmoid Epiploicae (3) Peritoneum (2) No Interposition (1) Other Tissues (4)
Matei et al. 2017 [19]		5	Transvesical (5)	—	No Interposition (5)
Bora et al. 2017 [13]		30	Transvesical (30)	—	No Interposition (12) Sigmoid Epiploicae (10) Omentum (6) Peritoneum (2)
Sanderson et al. 2018 [23]		5	Extravesical (5)	—	Sigmoid Epiploicae (5)
Körner et al. 2020 [28]		13	Extravesical (13)	—	No Interposition (12) Other Tissues (1)
Chandna et al. 2020 [22]		33	Transvesical (33)	Ureteric reimplantation (4) Burch colposuspension (1)	Sigmoid Epiploicae (16) No Interposition (9) Omentum (8)
Kumar et al. 2021 [8]		15	Transvesical (15)	Ureteric reimplantation (2) Ovarian cystectomy (1)	Omentum (13) Sigmoid Epiploicae (2)
Quadri et al. 2022 [26]		24	Transvesical (24)	Ureteric reimplantation (2)	Omentum (24)
Lecoanet et al. 2023 [25]	Transvesical	13	Transvesical (13)	—	Omentum (13) Peritoneum (5) Other Tissues (2) No Interposition (2)
	Extravesical	9	Extravesical (9)	—	
Yang et al. 2025 [20]		15	Transvesical (15)	Ureteric reimplantation (1) Cervical reconstruction (2)	Peritoneum (15)
Whole Review		206	Transvesical (169) Extravesical (37)	Ureteric reimplantation (13) Cervical reconstruction (2) Sigmoid colectomy (1) Burch colposuspension (1) Ovarian cystectomy (1)	Omentum (80) No Interposition (41) Sigmoid Epiploicae (38) Peritoneum (27) Other Tissues (15) Unknown (5)

± SD; (minimum – maximum); *IQR* Interquartile Range

### Outcomes

Combined mean OT was 169.5 ± 68.7 min in the TV group, 166.8 ± 74.1 min in the EV group, and 169.0 ± 69.5 min in the overall review. Comparison of the two technique subgroups showed no statistical significance (*p* = 0.857). EBL was minimal in all studies, with the highest mean EBL reported at 90 ml [21]. Overall POC rate was 4.37% and in the TV and EV group it was 3.55% and 8.11% respectively; however, the difference between the two subgroups was not statistically significant (*p* = 0.206). Major POC rate (defined as Clavien–Dindo grade III or IV) followed the same trend, with an overall rate of only 0.97% in the whole review and a higher rate in the EV group than in the TV approach group (2.70% vs 0.59% respectively); however, no statistically significant difference was found (*p* = 0.328). Overall recurrence rate was low at only 2.91%, and within each technique subgroup (2.37% with the TV and 5.41% in the EV approach); no statistically significant difference was found between subgroups (*p* = 0.294). Follow-up times varied substantially between studies, ranging from 1.5 to 35.3 months [8, 22, 23]. Hospital LOS was overall small, with 11 out of 14 studies reporting mean/median LOS under 5 days. Urethral CT varied substantially between studies, with values ranging from 10 to 25 days [20, 24]. The outcomes described are represented for each technique and in the whole review in Table 6 and the results of the statistical comparison between techniques in Table 7.

**Table 6:** Outcomes for each study and in the whole review

Reference		Mean OT ± SD (min)	Mean EBL (mL)	Mean LOS (days)	Mean CT (days)	POC rate, Clavien–Dindo I–IV (%)	Major POC rate, Clavien–Dindo III–IV (%)	Recurrence rate (%)	Mean follow-up (months)
Sundaram et al. [24]		233 ± 70	<70	5 (4–7)	10	0	0	0	6
Hemal et al. [21]		141 ± 16	90 (50–150)	3 (2–4)	14	0	0	0	(3–12)
Gupta et al. [9]		140 (110–180)	88 (50–200)	3.1 (2–5)	14–21	0	0	0	—
Gellhaus et al. [27]		249 (117–416)	53 (25–100)	2.1 (1–5)	—	10 (grade III, *n* = 1)	10 (grade III, *n* = 1)	0	28.3 (1–54)
Agrawal et al. [4]		215 ± 112	43 (5–100)	—	13 (9–16)	30 (grade I, *n* = 1; grade II, *n* = 2)	0	0	—
Matei et al. [19]		263 ± 41	—	6.6 (5–8)	14–21	0	0	0	—
Bora et al. [13]		133 ± 48	—	—	—	0	0	6.7 (*n* = 2)	—
Sanderson et al. [23]		218 ± 84	49 (30–70)	1.6 (1–3)	10–14	0	0	0	1.5
Körner et al. [28]		138 ± 59	—	—	10–14	7.7 (grade I, *n* = 1)	0	15.4 (*n* = 2)	4.6 ± 14
Chandna et al. [22]		165 ± 51	66	9.5 ± 4.6	21	0	0	6.1 (*n* = 2)	—
Kumar et al. [8]		126 (75–206)	32 (5–85)	4.4 (3–6)	18 (13–24)	0	0	0	35.3 (9–66)
Quadri et al. [26]		—	44	—	14–21	0	0	0	26 (5–52)
Lecoanet et al. [25]									
Transvesical		229 ± 70	—	—	14–21	23.1 (grade II, *n* = 2; grade III, *n* = 1)	7.7 (grade III, *n* = 1)	0	—
Extravesical		179 ± 77	—	—	10–15	11.1 (grade I, *n* = 1)	0	0	—
Yang et al. [20]		137 ± 45	14 (6–22)	4.9 (4.3–5.5)	25	0	0	0	—
Whole review		169.0 ± 69.5 (*n* = 143)	NA	NA	NA	4.37 (*n* = 9)	0.97 (*n* = 2)	2.91 (*n* = 6)	NA

*CT* catheterization time, *EBL* estimated blood loss, *LOS* length of stay, *NA* not available, *OT* operative time, *POC* postoperative complication, *SD* standard deviation

**Table 7:** Comparison of the outcomes of the two techniques

	Whole review	Transvesical	Extravesical	*p* value
Mean OT, min, mean ± SD	169.0 ± 69.5 (*n* = 143)	169.5 ± 68.7 (*n* = 116)	166.8 ± 74.1 (*n* = 27)	0.857
POC rate, %	4.37 (*n* = 9)	3.55 (*n* = 6)	8.11 (*n* = 3)	0.206
Major POC rate, ≥ Clavien–Dindo grade III, %	0.97 (*n* = 2)	0.59 (*n* = 1)	2.70 (*n* = 1)	0.328
Recurrence rate, %	2.91 (*n* = 6)	2.37 (*n* = 4)	5.41 (*n* = 2)	0.294

*OT* operative time, *POC* postoperative complication

## Discussion

Since the advent of RAS, there has been growing interest in applying this technology to VVF repair, especially given the limitations of conventional laparoscopy, namely the restricted operative field, challenging intracorporeal suturing, and a steep learning curve [7, 8]. To our knowledge, this systematic review and quantitative synthesis represents the most comprehensive synthesis to date, comprising outcomes of 14 studies evaluating robotics-assisted repair of VVFs. More than a third (35.9%) of the cases included in this review were recurrent fistulas, reflecting prior failed attempts at repair using other surgical techniques. Additionally, a substantial proportion involved complex fistulas or patients with a history of prior pelvic surgeries, where extensive adhesions were anticipated. These factors likely influenced the choice of RAS in the studies included, underscoring its utility in challenging clinical scenarios where conventional transvaginal or laparoscopic approaches may be technically unfeasible or less effective.

Regarding LOS, only three studies reported longer LOS than 5 days. Bora et al. [13] and Chandna et al. [22] (median of 7.5 and mean of 9.5 days respectively) contextualized these results with patient-related and local health care system factors, such as the majority of patients being housewives from remote areas, which meant that they were not restricted by formal employment obligations such as sick leave and the affordability of care. Matei et al. [19] (who reported a mean time of 6.6 days) also offered “geographic reasons” as a justification for their longer hospitalization times. When discarding these three outliers, LOS was relatively short (under 5 days), both in the overall review and within each technique subgroup. This trend toward shorter postoperative hospitalization aligns with the reduced morbidity and faster recovery typically associated with minimally invasive approaches, particularly robotics-assisted techniques.

The wide variation in reported CTs across studies reflects the heterogeneity of post-surgical care standards in the collected literature, particularly with regard to removal of the urinary catheter in these patients: some authors used a cystogram or a voiding cystourethrography to confirm the patency of the bladder closure before removing the catheter [4, 19, 23, 24]; others removed it “based on operating surgeons’ preference” [26]; and others routinely removed it after a certain postoperative period, which varied among studies [20, 21].

As described above, our study’s findings support the robotics-assisted repair of VVF as a safe and effective technique, given its low recurrence rate coupled with minimal EBL, short hospital stays and a low complication rate. However, one of the most frequently stated concerns regarding RAS is its potential for increases in OT, owing to setup and docking requirements [9, 30]. It has been well established in the literature that TA minimally invasive approaches are associated with a longer OT than the TV route [31]. However, it is less clear if the robotics-assisted repair of VVF involves, in fact, a longer OT than the other TA alternatives (conventional laparoscopy and laparotomy). Evidence from Gupta et al. [9] demonstrates that OTs are comparable between RAS and open abdominal surgery. In their retrospective comparative study of recurrent VVF repair, the mean OT was 140 min for robotics-assisted repair and 148.5 min for open repair, with no statistically significant difference observed (*p* > 0.05). However, given the small sample size (*N* = 32) and retrospective nature of the study, the authors concluded that, regarding this specific point, future comparative prospective studies with larger population pools are needed to clarify further [9]. With respect to conventional laparoscopy, no comparative studies were conducted comparing the OT of RAS and this technique. The present review’s overall mean OT of 169.0 ± 69.5 min is longer than some of the largest previously published case series using only conventional laparoscopy, such as those by Miklos and Moore [32] (*n* = 44), Sharma et al. [33] (*n* = 22), and Tilala et al. [34] (*n* = 36), which reported mean OTs of 144.8, 140, and 128.5 min respectively. That said, some of the longer mean OTs reported in the present review have been attributed by authors to factors such as limited surgeon experience—as in the initial series of five patients by Sundaram et al. [24] (233 min)—or the performance of concomitant procedures, as described by Agrawal et al. [4] (214 min), where three patients underwent ureteral reimplantation and one required a sigmoid colectomy [4, 13]. Furthermore, some authors point to other variables that could significantly impact OT, such as non-uniformity in reporting methodology, as few doctors record the console time [26], the use or omission of tissue interposition, and the type of interpositional tissue employed [23]. Another important factor is the surgical technique adopted for VVF repair. An increasing trend has been observed toward the use of a modified O’Conor technique—the Mini O’Conor—which entails a limited posterior cystotomy rather than full-bladder bivalving. This approach has been associated with reduced OT, as reported by Hemal et al. [21]. In conclusion, in order to definitively state that robotics-assisted VVF repair entails longer OT than conventional laparoscopy, future comparative studies need to control for the aforementioned confounding factors.

Robotics-assisted surgical repair of VVF may serve as a particularly valuable approach in the management of recurrent cases, which are often surgically challenging owing to increased technical complexity. Two case series focused specifically on recurrent fistulas [9, 21]; in total, these included 19 patients with a total of 25 previous attempts at fistula repair (16 TA and 9 TV), given that some patients underwent multiple previous attempts. Recurrence rate was 0% in both studies; however, although Hemal et al. [21] reported a follow-up period of 3–12 months, Gupta et al. [9] did not report on their follow-up period. Both studies achieved this result with a low EBL (means of 90 and 88 ml respectively), a POC rate of 0%, short mean hospital stays (3 and 3.1 days respectively), and mean OT inferior to the review’s overall combined mean (141 and 140 min versus 169 min) [9, 21]. As previously mentioned, Gupta et al. [9] also compared outcomes of RAS with a matched control group of patients who underwent open repair for recurrent VVF in their retrospective study. Their findings demonstrated that RAS was associated with reduced blood loss (88 ml vs 170 ml; *p* < 0.05) and shorter hospital stays (3.1 days vs 5.6 days; *p* < 0.05), while achieving comparable success rates and OTs. These results suggest that RAS might be a safe and effective option for the repair of recurrent VVF and might offer advantages over open surgery in this context. However, to date, and to our knowledge, no comparative studies have evaluated RAS against conventional laparoscopy in this specific patient subgroup.

In established laparoscopic series, Miklos and Moore [32] reported a mean OT of 144.8 min and a mean EBL of 51 ml following laparoscopic EV VVF repair, with a short mean hospital stay of 1.2 days, no major POC, and a 98% success rate at a mean follow-up of 17.3 months. Similarly, Tilala et al. [34], in a comparative analysis of laparoscopic TV and EV repairs, observed OT of 117 and 140 min respectively, with greater blood loss (up to 210 ml) and longer hospitalization associated with the TV approach, while reporting success rates of 94.45% and 83.33% at mean follow-up durations of approximately 13–15 months. Differences between robotics-assisted and laparoscopic outcomes likely reflect the heterogeneity in case selection, including the proportion of recurrent and complex fistulas in robotics-assisted series, as well as variation in perioperative protocols and follow-up duration. Overall, available evidence suggests that robotics-assisted VVF repair achieves outcomes comparable with well-established laparoscopic techniques, although the predominance of retrospective studies and heterogeneous reporting limits definitive conclusions regarding comparative effectiveness. POC profiles were similarly favorable across approaches. Neither Miklos and Moore [32] nor Tilala et al. [34] reported any major POC (Clavien–Dindo III–IV) in their laparoscopic cohort. These findings mirror the low major complication rates reported in robotics-assisted series, supporting the safety of both minimally invasive approaches. Finally, regarding success outcomes, Miklos and Moore [32] reported a similar success rate (98%) to the one demonstrated in the present review (98%), whereas Tilala et al. [34] reported a lower success rate in the TV group (83.33%) than in the EV group (94.45%), although the difference was not statistically significant. Differences in reported success rates may reflect heterogeneity in follow-up duration, inclusion of recurrent or complex fistulas, and variability in definitions of success. Overall, although OT may be longer in robotics-assisted series, perioperative morbidity and fistula closure outcomes appear comparable with established laparoscopic techniques. However, both laparoscopic and robotics-assisted studies predominantly consist of small, retrospective cohorts with variable case complexity and surgeon experience, which limits direct comparison and precludes definitive conclusions regarding the superiority of one minimally invasive approach over another. Taken together, these findings suggest that robotics-assisted VVF repair offers outcomes comparable with established laparoscopic techniques, with potential advantages related to ergonomics and suturing dexterity, although current evidence remains insufficient to support routine adoption over laparoscopy in the absence of prospective, comparative, and cost-effectiveness data.

The transvaginal approach has been consistently associated with primary closure rates of 82–98% across multiple single-center series [35–39], with higher success rates reported as surgical experience increases. Regarding its comparison with RAS in the treatment of VVF, a recent systematic review and meta-analysis by Srivastava et al. [40] specifically analyzed transvaginal repair in a series of surgically challenging cases, such as supratrigonal, juxtacervical, vault, and apical VVF, and reported a pooled success rate of 86.3%, notably lower than the 97.1% success rate observed in the present review of robotics-assisted repairs (defined as lack of recurrence in each follow-up period). These findings suggest that although the transvaginal approach may be highly effective in selected patients, cases involving certain fistula characteristics, such as high-lying or complex ones, may potentially benefit from a TA, and specifically robotics-assisted, approach. Nonetheless, further prospective and comparative evidence is required to establish the optimal surgical route in such patient subgroups.

The findings of this review are consistent with those reported by Lecoanet et al. [25], who conducted the first retrospective multicenter study comparing robotics-assisted TV and EV approaches to VVF repair. Both studies demonstrated no statistically significant differences between the two techniques across multiple outcomes, including OT, POC rate, and recurrence rate. Although not reaching statistical significance in either study, there was a tendency toward shorter OTs in the EV group, which may reflect the less invasive nature of this approach, as it avoids bladder bivalving and allows for more limited dissection. Conversely, both the present review and the study by Lecoanet et al. [25] observed a numerically higher rate of POCs in the EV group, including one case of ureteral injury in their series. This could be explained by the greater technical challenge of dissecting the vesicovaginal space without direct visualization of the bladder interior or ureteral orifices, particularly in patients with prior pelvic surgery. These procedural differences may account for the subtle trends observed in perioperative outcomes, despite the overall similarity in clinical efficacy between the two techniques. These findings are in accordance with previous evidence of surgical outcome similarity between both approaches in the context of laparoscopic repair of VVF: a systematic review in 2015 by Miklos et al. [5] compared the EV and TV approaches in the context of minimally invasive TA surgery (only 12.84% were RAS) and also found no statistically significant difference in outcomes between techniques. Since then, a recent (2024) comparative analysis study by Tilala et al. [34] comparing the two techniques in a conventional laparoscopy setting, found the EV technique to be associated with significantly shorter OT, lower EBL, reduced LOS, and fewer POCs. Although this study was not randomized and did not utilize RAS, its findings suggest that some of the outcome trends observed in the present review, specifically regarding OT, might reach statistical significance as further comparative evidence becomes available. Nevertheless, given the current evidence, it is our view that the choice between an EV or TV approach to VVF repair should primarily be guided by the surgeon’s experience and familiarity with the technique. That said, patient- and fistula-specific factors, such as a history of prior pelvic or fistula surgery, as well as fistula location near the ureteric orifices, should also be taken into account, given that in these situations, the TV approach could offer an easier and potentially safer pathway, avoiding ureteric injury. This is illustrated by the findings of Zhou et al. [10], who compared laparoscopic EV and TV approaches in a cohort of patients with dense adhesions between the bladder and vagina; in their study, OTs were significantly longer in the EV group (*p* < 0.001), suggesting that the TV technique might have been more suitable for these patients.

Despite the promising results observed in this review, the widespread implementation of RAS for VVFs remains limited, particularly in low-resource settings. In these regions, where the burden of obstetric fistulas is highest, access to robotics-assisted platforms is virtually non-existent owing to infrastructure limitations, cost constraints, and a lack of trained personnel [41, 42]. This is especially relevant given that the majority of VVFs in these underserved regions are still of obstetric origin, which are typically larger, more complex, and associated with significant tissue loss owing to prolonged obstructed labor [43, 44]. Additionally, there is to our knowledge currently no large-scale cost-effectiveness analysis published for robotics-assisted repair of VVF, despite the well-recognized high capital and maintenance costs associated with RAS [21, 25]. Although robotics-assisted approaches may reduce morbidity, hospital stay, and potentially long-term costs by minimizing complications and recurrence, these theoretical benefits must be weighed against the substantial upfront investment. This economic factor remains a significant barrier to broader adoption, particularly in public health systems and resource-constrained environments. Future research should explore not only clinical outcomes (particularly in comparison with conventional laparoscopy) but also cost-effectiveness metrics to better inform policy decisions and global applicability.

This review has some limitations, such as the fact that most included studies were retrospective case series with small sample sizes, which may introduce selection and reporting biases. There was considerable heterogeneity among studies regarding surgical techniques, patient characteristics, and outcome definitions, as well as small sample sizes, particularly in the EV group. Furthermore, the absence of long-term follow-up in many cases limits the ability to assess the durability of fistula repair over time. Several potentially relevant outcomes, including EBL, LOS, and urethral CT, could not be statistically compared owing to incomplete or inconsistent reporting of summary statistics. Although methods exist to estimate means and SDs from medians and associated measures of dispersion, these were not applied given the small study sizes and substantial heterogeneity across studies, and this represents an important limitation of the present review. Last, publication bias cannot be excluded, as studies with negative outcomes may be underrepresented in the literature.

## Conclusions

In this systematic review, the most comprehensive to date, robotics-assisted repair of VVFs appears to be safe and effective in selected patients, with low reported complication and recurrence rates, minimal blood loss, and short hospital stays. However, the available evidence is predominantly derived from small, retrospective case series with heterogeneous reporting and limited follow-up, which limits the strength of these conclusions. Although no statistically significant differences were observed between TV and EV robotics-assisted approaches, the overall quality of evidence remains low. Further prospective, comparative, and cost-effectiveness studies are required before routine adoption of robotics-assisted VVF repair can be recommended.

## Data Availability

No new datasets were generated or analyzed in this study. All data are derived from previously published studies, which are cited within the article.

## Abbreviations

BMI: Body mass index
CT: Catheterization time
EBL: Estimated blood loss
EV: Extravesical
JBI: Joanna Briggs Institute
LOS: Length of stay
OT: Operative time
POC: Postoperative complication
PRISMA: Preferred Reporting Items for Systematic Reviews and Meta-Analyses
RAS: Robotics-assisted surgery
SD: Standard deviation
TA: Transabdominal
TV: Transvesical
VVF: Vesicovaginal fistula
